# Accuracy of Three-Dimensional (3D) Printed Dental Digital Models Generated with Three Types of Resin Polymers by Extra-Oral Optical Scanning

**DOI:** 10.3390/jcm10091908

**Published:** 2021-04-28

**Authors:** Eugen S. Bud, Vlad I. Bocanet, Mircea H. Muntean, Alexandru Vlasa, Sorana M. Bucur, Mariana Păcurar, Bogdan R. Dragomir, Cristian D. Olteanu, Anamaria Bud

**Affiliations:** 1 Faculty of Dental Medicine, University of Medicine and Pharmacy, Science and Technology George Emil Palade, 540139 Târgu-Mureș, Romania; Eugen.bud@umfst.ro (E.S.B.); marianapac@yahoo.com (M.P.); Anamaria.bud@umfst.ro (A.B.); 2 Technical University of Cluj-Napoca, 400114 Cluj-Napoca, Romania; vlad.bocanet@tcm.utcluj.ro (V.I.B.); mircea.muntean@spectromas.ro (M.H.M.); 3 Faculty of Medicine, University Dimitrie Cantemir, 540545 Targu-Mures, Romania; bucursoranamaria@gmail.com; 4 Faculty of Dental Medicine, University of Medicine and Pharmacy Grigore T. Popa, 700115 Iași, Romania; 5 Faculty of Dental Medicine, University of Medicine and Pharmacy, 400010 Cluj-Napoca, Romania; olteanu.cristian@umfcluj.ro

**Keywords:** 3D printed dental models, polymer resin, extra-oral scanning

## Abstract

Digital impression devices are used alternatively to conventional impression techniques and materials. The aim of this study was to evaluate the precision of extraoral digitalization of three types of photosensitive resin polymers used for 3D printing with the aid of a digital extraoral optical scanner. The alignment of the scans was performed by a standard best-fit alignment. Trueness and precision were used to evaluate the models. The trueness was evaluated by using bias as a measure and the standard deviation was used to evaluate the precision. After assessing the normality of the distributions, an independent Kruskal–Wallis test was used to compare the trueness and precision across the material groups. The Mann–Whitney test was used as a post-hoc test for significant differences. The result of the analysis showed significant differences (U = 66, z = −2.337, *p* = 0.019) in trueness of mesiodistal distances. Upon visual inspection of the models, defects were noticed on two out of nine of the models printed with a photosensitive polymer. The defects were presented as cavities caused by air bubbles and were also reflected in the scans. Mean precision did not vary too much between these three photosensitive polymer resins, therefore, the selection of 3D printing materials should be based on the trueness and the required precision of the clinical purpose of the model.

## 1. Introduction

In-office dental 3D printing helps improve the efficiency of forward-thinking practices all over the world. By leveraging existing technologies that exist in digital dentistry, 3D printing enables better responsiveness to patient needs, significantly reduces manufacturing times, and opens up new treatment options. With low operating costs, minimal maintenance, and user-friendly design, these products make it easy to bring digital dentistry and 3D printing together in dental practice. 3D printing dental models and digital wax-ups reveal anatomical details, high precision for exceptional measurements, patient education, and dental laboratory collaboration. A large selection of dental 3D printing materials are designed, developed, and tested for high performance in digital dentistry. 3D printing resins are built to achieve equivalent or superior results than conventional dental materials while providing better value for money [1]. Digital impression making using intraoral and extraoral scanners may be an approach to improve the accuracy of dental restorations, as, by their nature, these processes tend to eliminate the error caused by conventional impression making and gypsum model casting [2]. There are only a few studies published on the accuracy of printed models compared with plaster models [3,4,5,6]. These studies concluded that the printed models can be used as a replacement for plaster models, but it is unclear whether the samples used in these studies were sufficient to draw definitive conclusions. Given 3D printing’s promising potential and increased use in dentistry, it is essential to evaluate the accuracy of 3D printed dental models [7]. An accurate printed model is fundamental for dental diagnostic purposes. 

With the introduction of computer-aided design (CAD) and computer-aided manufacturing (CAM) technologies in dentistry, virtual models of teeth are required. Digital processes are applied for prosthetic-driven backward planning of implant surgery, orthodontic measurements, and treatment planning combined with surgical planning. Data acquired by intraoral scanning, computed tomography, cone-beam computed tomography, and extraoral surface scanning can be fused [8,9,10]. Digital three-dimensional (3D) models are created by scanning impression and plaster models using desktop scanners or otherwise by cone-beam computed tomography. These methods have been widely accepted in clinical orthodontics and are advantageous due to the compact storage space, their potential to expand applications for treatment planning, and their easy customization [11,12].

The majority of the literature has focused on the reproducibility error of obtaining the 3D datasets either indirectly via sequential dental models [13,14,15,16] or directly using digital scanners when analyzing the accuracy of the 3D modes. However, the superimposition or alignment of the two datasets is not trivial and is also prone to error [17]. The alignment of the scans is performed by minimizing the mesh distance error between each corresponding data point. In our study, a standard best-fit alignment [18] was used. This method uses an iterative closest point (ICP) algorithm to align scans, with each software using a slightly different algorithm and does not involve operator-based decisions. The alignment is performed by minimizing the mesh distance error between each corresponding data point [18,19,20].

The precision of a 3D Printer NextDent™ using three different photosensitive polymer resins for three-dimensional (3D) printings with the help of GOM ATOS Capsule™ structured light optical scanner was examined in this study.

## 2. Materials and Method

A power study assuming 80% power and an alpha of 0.05 showed that 3 pairs of printed models for each material group were needed to show statistical differences of 0.5 mm in measurements with a 0.2 mm standard deviation [21].

**Figure 1:**
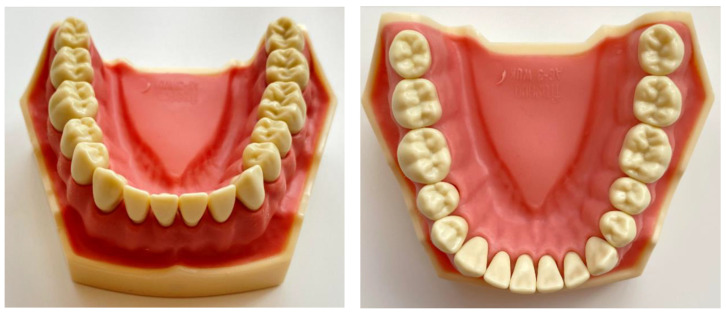
Model used for the reference comparison.

A typodont model (Frasaco™ Gmbh, Tettnang, Germany) containing 16 mandibular permanent teeth (Figure 1) was chosen as a reference model. With the aid of GOM ATOS Capsule (Zeiss™ Gmbh, Braunschweig, Germany), which is an optical precision measuring machine, the reference model was scanned. This device used 2 12Mp CCD cameras and a fringe blue light projector to scan the surface. Spatial referencing was done via uncoded markers, while the stereo camera technology provided an overdetermined system of equations for each measurement. It was able to measure the reference markers with a deviation of 3 µm to 5 µm. Its result was a 3D mesh created by polygonizing the large number of triangulated points captured by the cameras. The scan of the reference model was used as a benchmark for comparison later in the study.

Three photosensitive resins were used in the study from 3 different producers. The name and brand of producers were intentionally omitted because of commercial purposes and were generally named Producer 1, Producer 2, Producer 3. The material from Producer 1 had a price range of 300$ to 400$ per 1 kg container, Producer 2 from 200$ to 300$ per 1 kg container, and Producer 3 under 100$ per 1 kg container. Three models were printed from each material using the reference model scan. 

A NextDent 5100 (Soesterberg, The Netherlands) 3D printer was used, having the following settings: Build volume 124.8 × 70.2 × 196 mm (4.9 × 2.8 × 7.7 in), resolution 1920 × 1080 pixels, pixel pitch 65 microns (0.0025 in) (390.8 effective PPI), wavelength 405 nm. This printer used light (wavelength of 405 nm) to cure the resin. The resulting 9 models were scanned with the same 3D scanner as the reference model in similar light and temperature conditions. An observer repeated the measurements at a 1-week interval. As the model made by Producer 3 was shinier, the decision was made to cover the models with an antireflective powder. To preserve the measurement conditions, all models were covered irrespective of the material. The meshes resulting from scanning were exported in STL format. The STL file resulting from the scan of the reference model was used for printing the models from the 3 materials.

**Figure 2:**
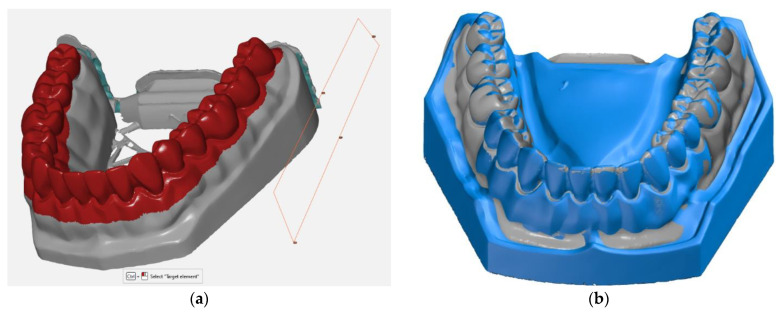
(**a**) Local best-fit alignment done in the region of interest between the 3D printed model and the reference surface; (**b**) the scanned surface (grey) and the reference surface (blue) overlapped.

Later in the study, the meshes of models were compared with the reference scan, and distances were measured using the GOM Inspect 2020™ (Braunschweig, Germany) software package. The printed model and the reference model surfaces were pre-aligned through a standard best fit method. This method globally minimizes the deviations between the 2 surfaces. As this method only superimposes the 2 surfaces to get a globally acceptable deviation, a local best fit method was used to align the 2 surfaces in the teeth area (marked with red in Figure 2). This method minimized the deviations between the 2 entities in the region of interest for this study.

**Figure 3:**
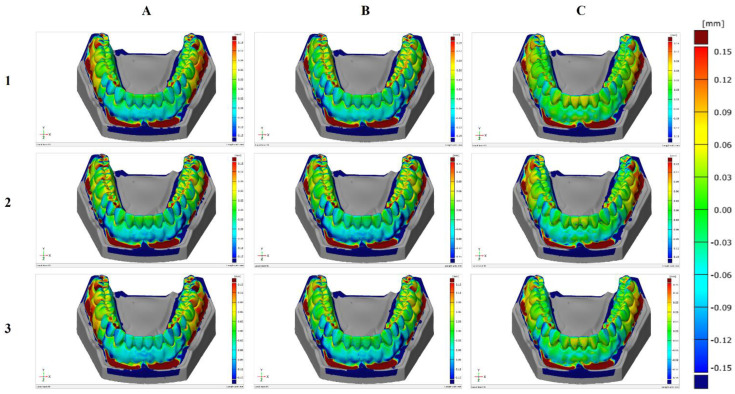
Surface comparison of the three printed models with the reference model for each material: (**A**) Producer 1; (**B**) Producer 2; (**C**) Producer 3.

In the subsequent stage of the study, a comparison between the 3D printed model surface and the reference model surface was performed, outlining the deviations between them. Figure 3 shows these comparisons for each model grouped by material. Positive deviations were noticeable in the molar regions (yellow and red), while negative deviations can be visible in the incisor and canine regions (blue). On average, these deviations ranged from −0.06 to 0.06 mm.

**Figure 4:**
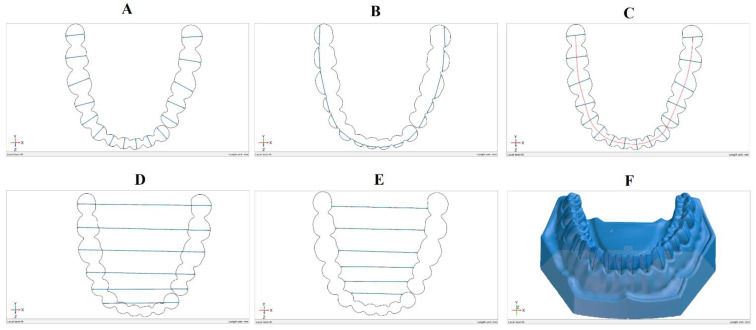
Measurements done on the models: (**A**) The buccolingual width, (**B**) the mesiodistal width, (**C**) the arch curve length, (**D**) arch width (exterior), (**E**) arch width (interior).(**F**) 3D printed model surface

For each model, the buccolingual width and mesiodistal width of each tooth were measured and the length of the arch curve. The dental arch width was also measured (the inter-canine, inter-premolar, and inter-molar distances) from the interior and exterior surfaces of each tooth and the height of each tooth (Figure 4).

**Figure 5:**
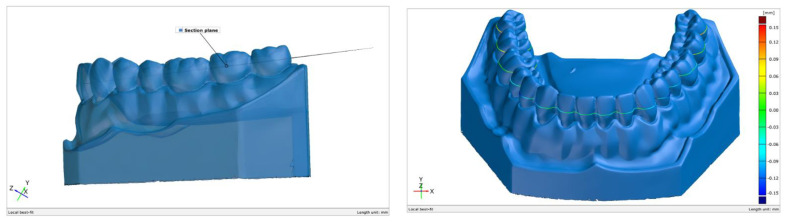
Section plane used for the buccolingual width measurements.

The buccolingual tooth width was measured using a section plane to better quantify the deviations between the models (Figure 4A). To obtain the section plane, the crown heights of molars 38, 48, and incisor 41 were measured. The midpoints of the distances from the mucogingival junction to the occlusal surface of each of these 3 teeth were used to create the plane (Figure 5).

**Figure 6:**
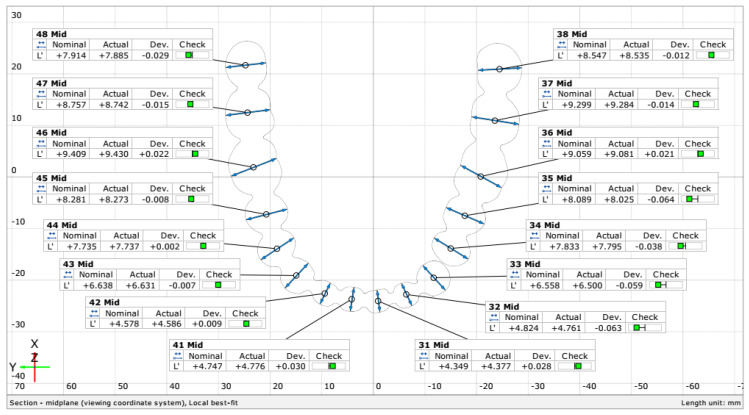
Section measurements in the buccolingual direction for each tooth.

Surface points were placed on opposite sides of each tooth in the buccolingual direction, and the distance between the points was measured (Figure 6). Repeatability was ensured by using the same measurement program for all models.

The mesiodistal distance (Figure 4B) was more difficult to determine because of the way the model was scanned and printed. The stereoscopic scanner that was used was not able to scan until the point where the teeth touch on the real reference model. As a result, the gaps in the mesh were filled, creating a continuous surface. An exception was the distance between incisor 33 and premolar 34 that was wide enough to permit the creation of 2 distinct surfaces on the reference model. The mesiodistal distance was determined such that it is on either side of the measured tooth but not in the filling area from between the teeth. Measurements were made only in the midplane created for the buccolingual measurements.

The arch curve was determined through the midpoints of the buccolingual distance of each tooth on the midplane (Figure 4C). 

The dental arch width was determined by measuring the inter-canine, inter-premolar, and inter-molar distances both from the exterior and interior surfaces of each tooth, as presented in Figure 4D,E. The height of each tooth was measured from the marginal gingiva to the occlusal surface of each tooth (Figure 4F).

Trueness and precision were used to evaluate each variable of the arch and tooth measurements. According to ISO 5725, trueness refers to “the closeness of agreement between the arithmetic mean of a large number of test results and the true or accepted reference value” while “precision refers to the closeness of agreement between test results” [21]. Trueness is a measure of systematic error while the precision of random error [22,23,24]. The standard deviation was used to evaluate the precision, while bias was used to evaluate trueness:

$$\begin{array}{cc} s & =\sqrt{\frac{\sum_{i=1}^{n}{\left(x_{i}-\bar{x}\right)}^{2}}{n}}\\ ias & =abs\left(\bar{x}-x_{nom}\right) \end{array}$$

where: $x_{i}$ is the measured value on a model for a specific characteristic. $\bar{x}$ the mean of measurements for the 3 models printed from the same material. n=3 is the number of measurements. $x_{nom}$ is the nominal value from the reference model.

## 3. Statistical Analysis

The Shapiro–Wilk test was used to assess the normality of the distributions. An independent-samples Kruskal–Wallis test was used to compare the precision and trueness values across the material groups. In the cases of significant results, a Mann–Whitney test was performed between each group pair. The level of significance chosen was *α* = 0.05. The analysis was done in IBM SPSS v26, and the data were preprocessed in Microsoft Excel 365.

## 4. Results

**Figure 7:**
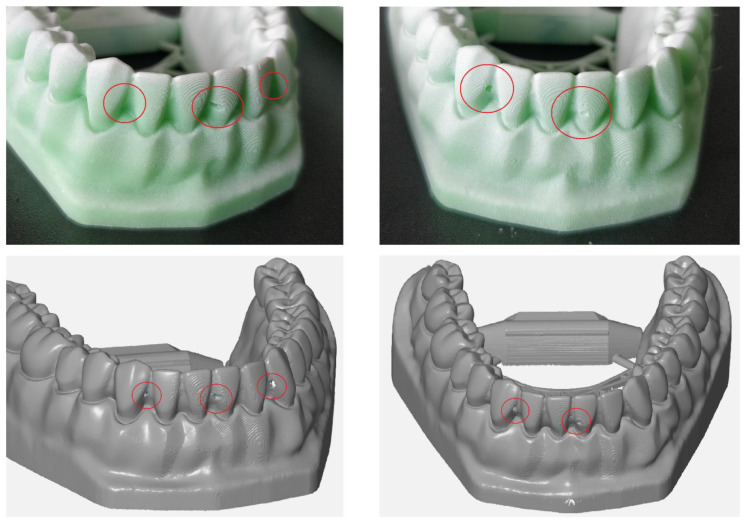
Defects, shown in red circles, in two models printed with the material from Producer 3 visible in the resulting scan.

Upon visual inspection of the models, defects were noticed on two out of three of the models printed with material from Producer 3. The defects presented as cavities caused by air bubbles. The defects were also reflected in the scans, as presented in Figure 7. As a result of the placement of these defects, some values of the mesiodistal distances were unable to be computed as the defects were in the exact region of the measurements.

**Figure 8:**
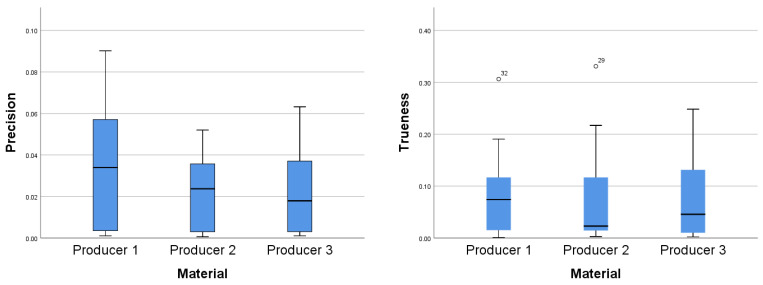
Distributions of precision values (**left**) and trueness values (**right**) for arch distances between the three material groups.

A Kruskal–Wallis test was conducted to determine if there were differences in precision and trueness values for arch distances between groups with materials from different manufacturers: Producer 1, Producer 2, and Producer 3. Distributions of precision values were not similar for all groups, as assessed by visual inspection of a boxplot (Figure 8). Although there were variations, median precision values were not statistically significantly different between the different material groups, for either precision (χ^2^(2) = 1.428, *p* = 0.490) or trueness (χ^2^(2) = 0.202, *p* = 0.904).

The differences in precision and trueness values for buccolingual distances between groups with materials from different manufacturers: Producer 1, Producer 2, and Producer 3 were assessed. Through visual inspection of the boxplot, it was concluded that the distributions of precision values were similar for all groups. Although there were variations, median precision values were not statistically significantly different between the different material groups, for either precision (χ^2^(2) = 2.327, *p* = 0.312) or trueness (χ^2^(2) = 4.349, *p* = 0.114). 

**Figure 9:**
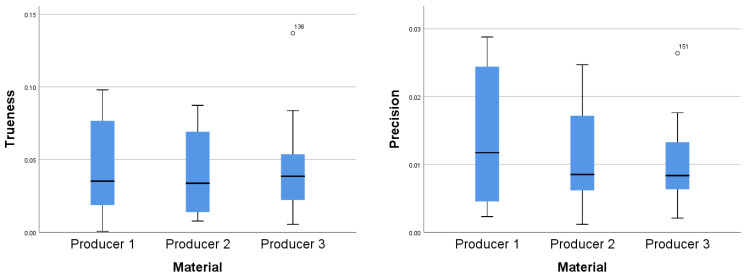
Distributions of precision values (**left**) and trueness values (**right**) for tooth height between the three material groups.

The same test was used to determine if there were differences in precision and trueness values for tooth height between groups with materials from different manufacturers: Producer 1, Producer 2, and Producer 3. Distributions of trueness values were similar for all groups but different for precision values, as assessed by visual inspection of the boxplots (Figure 9). Median precision values were not statistically significantly different between the different material groups, for either precision (χ^2^(2) = 0.391, *p* = 0.822) or trueness (χ^2^(2) = 0.145, *p* = 0.930). The precision and trueness mean (with the 95% CI) and standard deviations for the measurements are summarized in Table 1.

**Figure 10:**
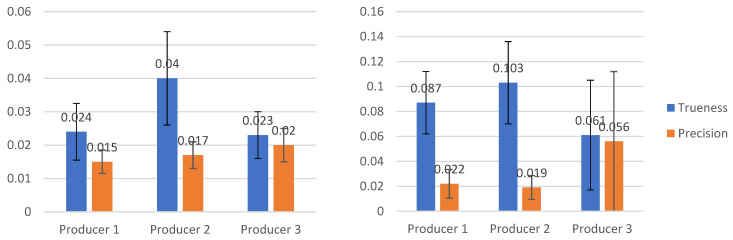
Buccolingual (**left**) and mesiodistal (**right**) measurements for the three material types.

The results for the buccolingual and mesiodistal measurements had a lower spread for precision than for trueness, except for the material from the third producer for the mesiodistal measurements (as seen in Figure 10).

The distributions of values for precision and trueness were tested for normality using both the Kolmogorov–Smirnov and the Shapiro–Wilk tests. The results of the tests were mixed, some values being normally distributed while others not. In addition, some outliers were identified. As a result, the decision was made to use non-parametric methods for further analysis as they do not make any assumptions regarding the distribution of data, and they are more robust to the presence of outliers than parametric methods.

Differences in precision values for the mesiodistal length between groups with materials from different manufacturers: Producer 1, Producer 2, and Producer 3 were assessed. Precision values were not distributed similarly for all groups, as assessed by visual inspection of a boxplot. Median precision values were statistically significantly different between the different material groups (χ^2^(2) = 6.370, *p* = 0.041. Subsequently, pairwise comparisons were performed using Dunn’s procedure with a Bonferroni correction for multiple comparisons. This post-hoc analysis revealed statistically significant differences in precision values between the material from Producer 1 and Producer 3 and between Producer 2 and Producer 3, but when applying the Bonferroni correction, the statistical significance disappeared. The decision was made to follow up with a Mann–Whitney test for the two pairs that resulted in a significant difference.

A Mann–Whitney U test was run to determine if there were differences in precision values for the mesiodistal distance between materials from Producer 1 and Producer 3. Precision values were statistically significantly higher for the material from Producer 3 (Mean rank = 20.38) than for the material from Producer 1 (Mean rank = 12.63), U = 66, z = −2.337, *p* = 0.019, using an exact sampling distribution for U [23].

The same test was run to determine if there were differences in precision values for the mesiodistal distance between materials from Producer 2 and Producer 3. Precision values were not statistically significantly different, U = 76, z = −1.960, *p* = 0.050. Although the precision values for the material from Producer 3 were higher (Mean rank = 19.75) than for the material from Producer 1 (Mean rank = 13.25), the p-value was marginally significant.

Distributions of the precision values were not similar, as assessed by visual inspection for either of the two tests.

Differences in trueness values for the mesiodistal length between groups with materials from different manufacturers: Producer 1, Producer 2, and Producer 3 were tested. After a visual inspection of the distributions of trueness values, it was determined that they were not similar. Median precision values were statistically significantly different between the different material groups, (χ^2^(2) = 10.050, *p* = 0.007. Adjusted p-values are presented. Subsequently, pairwise comparisons were performed using Dunn’s procedure with a Bonferroni correction for multiple comparisons. This post-hoc analysis revealed statistically significant differences in trueness values between the material from Producer 1 (Mean rank = 27.69) and Producer 3 (Mean rank = 15.56) (*p* = 0.043) and between Producer 2 (Mean rank = 30.25) and Producer 3 (0.009), but not between materials from Producer 1 and Producer 2 (*p* = 1.000). This shows that the material from Producer 3 has a significantly lower bias for the mesiodistal distances than materials from Producer 1 and Producer 2.

## 5. Discussion

In this study, printed models obtained from digital scans made with an extraoral scanner were used because extraoral scanning is increasingly used to make digital dental models, and some of the errors that can occur in the traditional impression-taking procedure can be avoided. Digital models have several advantages compared with plaster models, such as ease of data storage and data transmission, provide both visual and tactile information, and can be used for diagnostic, therapeutic, and education purposes. The goal of this study was to assess the trueness and precision of dental models obtained by the extraoral scanning technique, fabricated using three different types of polymer resin with a 3D printer. The accuracy of various 3D printed models has been validated only by a few studies. In one such study, Hazeveld et al. [6] decided to fabricate dental models using three types of rapid prototyping in order to analyze the accuracy of these models. They used digital calipers to measure the size of the teeth, focusing on measuring the mesiodistal height and width. It failed to measure the buccolingual width, which is also influenced by the method of printing and polymerization. This might have affected the fit of orthodontic appliances and individualized trays. In another study, Murugesan et al. [25] also made dental models using three types of rapid prototyping and also used digital calipers to measure the teeth in order to assess the accuracy of the models. The use of digital calipers in measuring the teeth might have led to errors in measurement due to the difficulty in finding the tooth of a reference point. Their models were printed by different printers, which might have also led to inconsistencies and errors in measurements. We tried to address both shortcomings by applying 3D software to validate the trueness and precision of the dental models made by the 3D printers and by establishing more clear reference points on the gingival areas and the teeth. We consider that the careful establishment of clear reference points played an important and decisive role in getting good results. Highly repeatable measurements have been reported by Salmi et al. [26], but in this study, they used a 10.0 mm reference point. 

Another important factor when scanning 3D printed models is represented by the scan spray (antireflective powder). This powder helps in lowering the reflection of light of the printed models. In the 3D color map of the experimental group, the labial and buccal surfaces of the 3D printed models in all experimental groups displayed a homogenous pattern of the blue area, which represented shrinkage. This might be explained by the characteristic of the surface in these areas, which was usually smooth and allowed the polymers to contact evenly. The opposite situation was present on the occlusal and interdental surface with its pit and groove regions, where an uneven pattern of shrinkage was observed.

As far as we know, the differences between 3D printed models and a reference model have never been carefully examined in order to prove they are clinically acceptable. One possibility might be that the dimension differences have no impact on clinical applications [3,27]. As specified in Hirogaki, Y [28] from a clinical perspective, a 0.3 mm dimension difference in dental models might be accurate enough. Depending on the treatment method, different clinical standards should be used for determining the accuracy and adequacy of dental models. The choice of 3D printing technology must be determined by its intended application. Hence, it is reasonable to conclude that 3D printed models, which are clinically acceptable for orthodontic purposes, may not necessarily be acceptable for the prosthodontic workflow or other dental applications requiring high accuracy [26,28].

When using 3D superimposition techniques, the risk of bias and applicability concerns are low as high accuracy desktop scanners are utilized, and CAM is the only identified source of error. However, it is worth noting that increased risk of bias and applicability concerns for index tests are recorded for studies that use linear measurements because of human error when performing physical linear measurements with no information provided on the calibration of the examiners. We believe that the dental models produced via 3D printing may be good enough for clinical purposes. It can be expected that the costs of printing dental models will decrease, and the costs will possibly become comparable with the conventional fabrication of plaster models. Increased use of CAD/CAM techniques for making customized orthodontic appliances with appliance printing techniques can be expected. However, in order to fully analyze the clinical efficacy and the accuracy of the 3D models, more studies are needed. 

## 6. Conclusions

There were significant discrepancies in the trueness of mesiodistal distance measurements between the 3D printing polymer resins. Producer 1 and Producer 2 were more precise than Producer 3 material, with the Producer 1 photosensitive polymer displaying the highest accuracy. Our results show that the material from Producer 3 has a significantly lower precision (a higher spread) for the mesiodistal measurements than the material from Producer 1. For the comparison between the materials from Producer 2 and Producer 3, the results are inconclusive as the p-value is marginally significant (*p* = 0.05). Mean precision does not vary too much between these three photosensitive polymer resins, therefore, the trueness and the required precision of the clinical purpose of the model should be deciding factors in choosing the proper 3D printing materials.

## Figures and Tables

**Table 1:** Descriptive statistics for precision and trueness for the three materials for each type of measurement.

Measurement	Indicator	Variable	Producer 1	Producer 2	Producer 3
Arch distances	Trueness	Mean (95% CI)	0.083 [0.042, 0.124]	0.073 [0.027, 0.119]	0.080 [0.042, 0.118]
		Std. Dev.	0.083	0.092	0.077
	Precision	Mean (95% CI)	0.033 [0.018, 0.048]	0.022 [0.013, 0.031]	0.024 [0.13, 0.034]
		Std. Dev.	0.030	0.018	0.021
Buccolingual measurements	Trueness	Mean (95% CI)	0.024 [0.015, 0.033]	0.040 [0.25, 0.55]	0.023 [0.015, 0.030]
		Std. Dev.	0.017	0.028	0.014
	Precision	Mean (95% CI)	0.015 [0.011, 0.019]	0.017 [0.012, 0.021]	0.020 [0.015, 0.026]
		Std. Dev.	0.007	0.008	0.01
Mesiodistal measurements	Trueness	Mean (95% CI)	0.087 [0.060, 0.113]	0.103 [0.067, 0.138]	0.061 [0.014, 0.108]
		Std. Dev.	0.050	0.066	0.088
	Precision	Mean (95% CI)	0.022 [0.009, 0.034]	0.019 [0.009, 0.029]	0.056 [−0.008, 0.120]
		Std. Dev.	0.023	0.019	0.112
Tooth height	Trueness	Mean (95% CI)	0.046 [0.029, 0.063]	0.043 [0.027, 0.058]	0.045 [0.027, 0.062]
		Std. Dev.	0.032	0.029	0.032
	Precision	Mean (95% CI)	0.014 [0.009, 0.019]	0.011 [0.007, 0.015]	0.010 [0.007, 0.013]
		Std. Dev.	0.010	0.007	0.006
Arch curve length	Trueness	Length	0.143	0.121	0.029
	Precision	Length	0.036	0.012	0.01
